# Face-Saving and Help-Seeking Among U.S. Chinese Older Adults With Elder Mistreatment

**DOI:** 10.1093/geroni/igab046.1654

**Published:** 2021-12-17

**Authors:** Ying-Yu Chao, Dexia Kong, XinQi Dong

**Affiliations:** 1 Rutgers University, Newark, New Jersey, United States; 2 The Chinese University of Hong Kong, Hong Kong , Hong Kong; 3 Rutgers University, Rutgers Institute for Health, New Jersey, United States

## Abstract

Chinese culture places a high value on saving face and not bringing shame to the family. This study aimed to examine the associations between face-saving and help-seeking among U.S. Chinese older adults who experienced elder mistreatment (EM). Data were retrieved from the PINE study. Regression analyses were performed. Most EM victims sought help from informal sources only (48.21%), followed by no help (26.79%), informal plus formal help (19.64%), and formal help only (5.36%). For EM screening, face-saving was associated with informal help-seeking intentions (p < .05). For EM subtypes, face-saving was associated with overall help-seeking intentions for financial exploitation (p < .05), but not on physical mistreatment, psychological mistreatment, and caregiver neglect. Face-saving was not associated with help-seeking behaviors. Study findings underscore the significance of a unique cultural value in understanding EM help-seeking intentions among Chinese older adults. Cultural constructs should be considered in future EM research in diverse populations.

